# Effectiveness of Some Natural Compounds Against Antibiotic-Resistant *Listeria* spp. and *Salmonella enterica* Strains

**DOI:** 10.3390/biology15141141

**Published:** 2026-07-13

**Authors:** David Jiménez-De-Juan, Félix Adanero-Jorge, Carlos Alonso-Calleja, Rosa Capita

**Affiliations:** 1 Department of Food Hygiene and Technology, Veterinary Faculty, University of León, E-24071 León, Spain; 2 Institute of Food Science and Technology (ICTAL), University of León, E-24071 León, Spain; 3 Instituto de Investigación Biosanitaria de León (IBIOLEÓN), Hospital de León, E-24071 León, Spain

**Keywords:** antibiotic-resistant bacteria, *Listeria* spp., *Salmonella enterica*, natural compounds, propolis, phenolic compounds, minimum inhibitory concentration, minimum bactericidal concentration

## Abstract

The increasing prevalence of antibiotic resistance highlights the need to seek alternatives to currently used compounds. Minimum inhibitory concentrations (MICs) and minimum bactericidal concentrations (MBCs) of nine ethanolic extracts of propolis (EEPs) from northwestern Spain and five phenolic compounds (carvacrol, eugenol, thymol, resveratrol, and trans-cinnamaldehyde) against *Listeria* spp. and *Salmonella enterica* strains were determined. All EEPs showed a strong antimicrobial effect against *Listeria* spp., with MICs (ppm) ranging between 78.00 ± 0.00 and 1041.67 ± 360.84 and MBCs (ppm) ranging between 312.50 ± 0.00 and 3333.33 ± 1443.38. The concentration of caffeic acid phenethyl ester (CAPE), isoprenyl caffeate (IPC), or total polyphenols did not influence the antimicrobial activity of EEPs. However, EEPs had only slight antimicrobial activity against *S. enterica*, with MICs ≥ 10,000 ppm and MBCs exceeding 10,000 ppm. MIC values (ppm) for phenolic compounds with respect to *Listeria* spp. strains ranged from 125.00 ± 0.00 to 2500.00 ± 0.00, and those for MBCs (ppm) ranged from 250.00 ± 0.00 to 5000.00 ± 0.00. For *S. enterica*, MICs (ppm) ranged from 250.00 ± 0.00 to 2500.00 ± 0.00 and MBCs (ppm) ranged from 416.67 ± 144.34 to 5000.00 ± 0.00. The results suggest that EEPs and phenolic compounds may offer natural alternatives to other antimicrobials in common use, with phenolic compounds being especially promising because of their antimicrobial activity against both Gram-positive and Gram-negative species.

## Simple Summary

Antibiotic resistance is a growing problem of great importance to public health. Therefore, it is important to seek alternatives to currently used antimicrobials. In this research, the efficacy of different natural compounds of plant origin against two bacterial species responsible for human foodborne infections (*Listeria* spp. and *Salmonella enterica*) was determined. Propolis ethanolic extracts are observed to be highly effective against *Listeria* spp., while phenolic compounds show variable efficacy depending on the compound and the microorganism. These results provide a starting point for the development of new antimicrobial compounds.

## 1. Introduction

Listeriosis is a zoonosis primarily transmitted through the consumption of contaminated foodstuffs. It affects certain risk groups more often and more seriously: the young, the old, the pregnant, and the immunocompromised—the so-called YOPIs [1]. Every year, there are approximately 23,000 cases of invasive listeriosis worldwide [2]. The European Union recorded a total of 3041 confirmed cases of invasive listeriosis in 2024 (0.69 cases per 100,000 population). These were associated with a mortality rate of 15.6%, the highest among all foodborne diseases [3]. Moreover, listeriosis infection causes major lesions and sequelae, such as meningitis, encephalitis, septicemia, endocarditis, and miscarriages [4]. The most important species in the genus *Listeria*, the etiological agent in the great majority of listeriosis cases and outbreaks in humans and animals, is *Listeria monocytogenes*. Other species (*Listeria ivanovii* and *Listeria seeligeri*) may also cause listeriosis on occasion [1,3].

*Salmonella enterica* is the salmonellosis-causing enterobacterium. Salmonellosis is the zoonosis associated with the greatest number of foodborne disease outbreaks in the European Union (1238 recorded in 2024). It ranks second to campylobacteriosis in terms of the number of human disease cases (79,703 in 2024), with a notified instance rate of 18.6 cases per 100,000 population [3]. Furthermore, *S. enterica* is the pathogenic microorganism implicated in most notifications to the EU Rapid Alert System for Food and Feed (RASFF). Specifically, in 2022, it triggered 603 notifications, followed by *L. monocytogenes* (132) [5]. The principal serotypes involved in human salmonellosis in the last few years in the EU were *S. enterica* serotype Agona, *S.* Enteritidis, *S.* Hadar, *S.* Infantis, *S.* Kentucky, *S.* London, *S.* Newport, *S.* Typhimurium, a monophasic variant of *S.* Typhimurium (4, [5],12:i:-), and *S.* Virchow [5,6,7].

In recent decades, there has been a marked increase in the prevalence of bacterial antibiotic resistance, a phenomenon involving principal pathogenic microorganisms and a broad range of antimicrobials [8]. It is estimated that by 2050, infections caused by resistant bacteria will be the leading cause of mortality worldwide, with some ten million deaths per year [9], a figure much higher than the 700,000 deaths attributable to antibiotic resistance recorded in 2014 [10]. The financial consequences of antibiotic resistance are also considerable, with an estimated annual cost of 1100 million euros to the health systems of the United States (USA) and the EU due to infections by resistant bacteria [11]. Although *L. monocytogenes* remains sensitive to most clinically relevant antimicrobials, an increase in the prevalence of antibiotic-resistant strains has been observed in recent years [1,9,12,13]. A high incidence of antibiotic-resistant and multi-resistant *S. enterica* isolates has been reported for years [14,15,16]. In this scenario, the treatment of both listeriosis and salmonellosis may be compromised.

The growing impact of antibiotic resistance suggests a need to seek alternatives to currently used compounds. A promising strategy involves using substances of plant origin, for example, propolis and phenolic compounds [17,18,19,20,21,22]. There is a lower risk of developing resistance to these natural substances than to antibiotics. This is because, unlike antibiotics, propolis and phenolic compounds, as plant terpenes or derivatives, act on multiple different points, with a consequent “multitarget” effect [19,20,21,22]. Moreover, some plant-derived antimicrobials have been approved by the United States Food and Drug Administration (FDA) as “Generally Recognized as Safe” (GRAS), indicating they are harmless and well accepted by consumers [23,24,25].

Propolis is a complex natural matrix of plant origin, comprising saps and resins of trees, bushes, and other flora that are processed by honeybees (*Apis mellifera*). It has a large content of bioactive organic substances that protect against pathogenic bacteria [22] and viruses (e.g., SARS-CoV-2) [23]. Its antimicrobial activity appears to be linked to various physiological processes, such as increasing permeability, disrupting cell membrane structure, modifying DNA synthesis, or blocking adenosine triphosphate (ATP) production. This multitarget effect damages several different locations and physiological processes in bacteria [22,26]. The composition of propolis from Northwest Spain has been recently studied [27]. Although the antimicrobial effectiveness of propolis and its extracts has been investigated by several authors [17,18,22], very little research has been conducted on propolis of Spanish origin, and the substance has yet to be tested against a collection of *Listeria* spp. and *S. enterica* strains. Thus, this research represents a novel and promising work in the search for new antimicrobials.

In terms of phenolic compounds, some of the most promising plant-derived antimicrobials are terpenes, such as carvacrol, thymol, eugenol, and trans-cinnamaldehyde, which are abundant in many plant essential oils. These substances are also sometimes found in propolis [17,18,27,28,29], and evidence suggests they have antimicrobial activity against various relevant pathogenic bacterial species, both Gram-positive and Gram-negative [19,20,28,29,30,31]. These compounds exert their antimicrobial activity through a multitarget mechanism, simultaneously affecting several vital bacterial structures and processes. A common and primary target is the cytoplasmic membrane, whose integrity and permeability are disrupted, leading to leakage of ions and intracellular contents [19,20,26,28,29,30]. Additionally, they can inhibit efflux pumps, known as “EPIs” (efflux pump inhibitors) [19,20,28,29,31,32,33], leading to an accumulation of phenolic terpene compounds within cells, and interfere with key enzymatic systems, such as ATPases and dehydrogenases, impairing energy metabolism and redox, leading to an accumulation of phenolic compounds within cells. This triggers a partial or total interruption of metabolism and progressive suppression of cell functions, culminating in cell death [19,29,30,31,34,35]. Notably, several phenolic terpenes also interfere with quorum-sensing systems, reducing the expression of virulence factors and biofilm formation in Gram-negative bacteria [19,20,26,28].

Another interesting compound is resveratrol. Although it is not a terpene but a stilbene-type phenolic compound with a sample structure, it also exhibits a multitarget antimicrobial profile. It interferes with nucleic acid synthesis, induces oxidative stress, and modulates gene expression related to virulence and stress response [29,30,31,32,36]. This diversity of targets reduces the likelihood of resistance development and supports the potential of natural phenolics as effective alternatives to conventional antibiotics.

This research aimed to determine the antimicrobial activity against antibiotic-resistant bacteria of nine propolis extracts from northwestern Spain and five phenolic compounds (carvacrol, eugenol, thymol, resveratrol, and trans-cinnamaldehyde) abundant in propolis and various essential oils. To this end, the minimum inhibitory concentrations (MICs) and minimum bactericidal concentrations (MBCs) of these substances were calculated with respect to 10 strains of *Listeria* spp. (nine *L. monocytogenes* and one *L. ivanovii*) and of 10 strains of *S. enterica* of different relevant serotypes.

## 2. Materials and Methods

### 2.1. Antimicrobial Substances Tested

Nine samples of raw propolis were selected and processed in accordance with the method proposed by Bonvehí and Gutiérrez [17], with slight modifications. The samples were gathered from nine different hives in various parts of the autonomous region of Castile and Leon in northwestern Spain, using pieces of food-safe plastic mesh measuring 42.5 cm by 51 cm. After eliminating impurities, aliquots of approximately 50 g were taken from each sample and stored in sterile polypropylene flasks of 150 mL capacity (Deltalab, Barcelona, Spain) at −20 °C for at least 24 h. Thereafter, the samples were milled in a laboratory grinder to produce raw propolis powder. For further experiments, 25 g from each milled sample was taken and stored in the dark at −20 °C in appropriately labeled plastic vials.

All the phenolic compounds tested (carvacrol, eugenol, thymol, resveratrol, and trans-cinnamaldehyde) were at least 95% pure and supplied by Sigma Aldrich Inc. (Saint Louis, MO, USA).

### 2.2. Working Solutions of Ethanolic Extracts of Propolis (EEPs) and Phenolic Compounds

From the raw propolis powder (see Section 2.1), natural liquid extracts in ethanol (Sigma Aldrich) were produced at a 70% concentration [18,22]. First, 2 g of frozen raw propolis powder was mixed with 20 mL of 70% ethanol, producing 10% (*w*/*v*) extracts. These extracts were kept under constant agitation in a mixer for 24 h at 25 °C to facilitate extraction of bioactive substances, such as polyphenolic compounds, terpenes, and alkaloids. They were then centrifuged for five minutes at 3200× *g* and passed through 0.22 µm filters (Millipore, Merck Kommanditgesellschaft auf Aktien, Darmstadt, Germany) using sterile 10 mL syringes (Omnifix^®^, B. Braun AG, Melsungen, Germany) to eliminate impurities and contaminants. After this filtering, the extracts were concentrated in a SpeedVac vacuum concentrator (Savant Instruments Incorporated, New York, NY, USA) at room temperature (approximately 20 °C) to eliminate ethanol. The extracts were kept at 4 °C in the dark until use. Immediately before determining the MICs and MBCs in the different assays, 10% working solutions were prepared in 70% ethanol, and from these initial 10% solutions, working extracts at lower concentrations were made in sterile distilled water (hydroethanolic extracts). The propolis samples were analyzed according to the techniques previously described [37]. For resin quantification, the Argentine standard for raw propolis [38] was used. The total determination of polyphenols was carried out by spectrophotometry at a wavelength of 760 nm, using the Folin–Ciocalteu reagent and acid gallic as the standard [37,38,39]. The total polyphenol content was expressed in gallic acid equivalents. Caffeic acid phenethyl ester (CAPE) and isoprenyl caffeate (IPC) contents were determined using Ultra Performance Liquid Chromatography (UPLC) [37,39].

For phenolic compounds, 10% ethanolic extracts were prepared in 70% ethanol several days before the experiments. All these solutions were kept at 4 °C in hermetically sealed glass flasks. Just like propolis extracts, from these initial 10% solutions, working extracts at lower concentrations were made in sterile distilled water (hydroethanolic extracts). Distilled water was used to avoid bacterial distortions due to the antimicrobial effect of alcohol (ethanol), and 7% ethanol was the maximum working concentration. In the case of eugenol and resveratrol, which are both less soluble in water, 1% Tween^®^ 80 (Sigma Aldrich) was incorporated into water to facilitate mixing and dissolving these compounds as [22,29,30,31,36,40,41,42]; this percentage (1%) is also allowed at the level of food safety set by the FDA [43]. All these hydroethanolic solutions contained 1–2% plant phenolic compound extracts and were ready for use in the MIC and MBCs determination assay (see Section 2.4).

### 2.3. Bacterial Strains and Antibiotic Resistance

Nine strains of *L. monocytogenes* were tested: ATCC (American Type Culture Collection) 19111 (serotype 1/2b), ATCC 19112 (serotype 1/2b), ATCC 19114 (serotype 4a), ATCC 19117 (serotype 4d), ATCC 13932 (serotype 4b), and STCC (Spanish Type Culture Collection) 936 (serotype 1/2b), STCC 937 (serotype 3b), STCC 938 (serotype 3c), and C6 (strain isolated from pork). Additionally, one *L. ivanovii* strain (ATCC 19119) was investigated, along with ten *S. enterica* strains isolated from poultry (serotypes *S.* Agona, *S.* Enteritidis, *S.* Hadar, *S.* Kentucky, *S.* Infantis, *S.* London, *S.* Newport, *S.* Typhimurium, *S.* Typhimurium monophasic variant 1, 4, [5],12:i:-, and *S.* Virchow).

The bacteria were screened on Mueller–Hinton agar (MHA, Oxoid) for susceptibility to a panel of ten antibiotics using the disc-diffusion method, as described in the Clinical and Laboratory Standards Institute (CLSI) guidelines [44]. The following discs were used for *Listeria* spp.: ampicillin (AMP, 10 μg), amoxicillin/clavulanic acid (AMC, 30 μg), penicillin (P, 10 units), trimethoprim/sulfamethoxazole (SXT, 25 μg), gentamicin (CN, 10 μg), erythromycin (E, 15 μg), ciprofloxacin (CIP, 5 μg), tetracycline (TE, 30 μg), vancomycin (VA, 30 μg), and rifampicin (RD, 5 μg). The antibiotics tested for *S. enterica* were as follows: ampicillin (AMP, 10 μg), amoxicillin/clavulanic acid (AMC, 30 μg), ceftriaxone (CRO, 30 μg), azithromycin (AZM, 15 μg), trimethoprim/sulfamethoxazole (SXT, 25 μg), ciprofloxacin (CIP, 5 μg), chloramphenicol (C, 30 μg), meropenem (MEM, 10 μg), imipenem (IMP, 10 μg), and fosfomycin (FOS, 200) [44,45]. All antibiotic discs were purchased from Oxoid. After incubation at 37 °C for 18 to 24 h, the inhibition halos were measured, and the strains were classified as susceptible, with reduced susceptibility (intermediate), or resistant, in accordance with the criteria of the CLSI [44] and the European Committee on Antimicrobial Susceptibility Testing (EUCAST, Linköping, Sweden) [45].

### 2.4. Determination of Minimum Inhibitory Concentrations (MICs) and Minimum Bactericidal Concentrations (MBCs) of Natural Compounds

To calculate the MIC and MBC values of each substance, five colonies of the given microorganism were taken from tryptone soy agar (TSA, Oxoid Ltd., Basingstoke, Hampshire, UK) plates, inoculated into test tubes holding 10 mL of tryptone soy broth (TSB, Oxoid Ltd.), and then incubated for 24 h at 37 °C until the stationary phase. Prior experiments showed that these cultures contained approximately 10^8^ CFU/mL after 24 h of incubation at 37 °C. This estimation was based on repeated plate count validations in our laboratory and the use of the 0.5 McFarland turbidity standard, a widely accepted method for standardizing bacterial inoculum density. Thereafter, 1:2 serial dilutions of these natural extracts were produced in microtiter plate wells (Deltalab S.L., Barcelona, Spain) at a range of concentrations through the Y axis of the microtiter plate.

The first well received 100 μL of extract, the maximum starting concentration, while the remaining wells each received 50 μL of TSB (1×). Thereafter, 50 μL of the contents of each well were transferred to the next in sequence, so that each well contained half the concentration of extract present in the previous well. Finally, 50 μL of the third 1:10 serial dilution of the respective strain tested (approximately 10^5^ CFU/mL; dilutions performed in TSB) was added to each well to a volume of 100 microliters. The contents of the wells (100 μL in total) were mixed with an automatic pipette [45].

Specifically, a concentration range of 10,000 to 78 ppm was used for the propolis extracts and eugenol, and a range of 2000 to 7.8 ppm was used for carvacrol, thymol, and trans-cinnamaldehyde, following a two-fold (1:2) dilution technique. Ethanol was used to dissolve resveratrol because of its limited solubility in water. Therefore, the maximum concentration tested for resveratrol was 1000 ppm since the antimicrobial activity of ethanol would mask the effects of resveratrol, as noted above [30,34,41].

It is worth noting that the concentration series was not designed arbitrarily but was based on preliminary solubility tests and literature data on the antimicrobial activity of natural compounds. For example, reported MIC values for ethanolic propolis extracts range from 117 to 1840 µg/mL for Gram-positive bacteria and from 34 to 5000 µg/mL for Gram-negative bacteria [18,22,46]. Terpenes such as thymol and carvacrol have MICs between 125 and 500 ppm [24,31,32,33,35], while resveratrol has MICs between 125 and 1000 ppm depending on the pathogen [29,30,34]. These values support the use of a 2-based dilution series ranging from 10,000 to 78 ppm for propolis and eugenol, from 2000 to 7.8 ppm for other terpenes, and up to 1000 ppm for resveratrol.

Four types of controls were included in this experiment, including (1) negative TBS controls to confirm the sterility of the culture broth, (2) controls for turbidity (TSB and the antimicrobials tested) to establish the limit of turbidity needed to consider that bacterial growth had occurred, and (3) positive controls (TSB inoculated with the strain) to check the growth capacity of the microorganisms.

Also, to avoid interference from the solvents, controls with 7% ethanol and 7% ethanol + 1% Tween^®^ 80 were tested separately. I was previously observed in our laboratory that these concentrations (maximum working concentration used) did not affect bacterial growth, but the results were considered under the same experimental conditions and times (see next paragraph).

In addition, a new category of controls was implemented, (4) solvent controls, which included TSB with the mentioned solvents (7% ethanol and 7% ethanol + 1% Tween^®^ 80) at the same concentrations used in the experiment (in this case, without natural antimicrobials) with the same dilution rate (1:2). The same testing plates and strains were used to confirm that these substances alone did not inhibit bacterial growth (all strains grew in different wells).

Finally, the plates were sealed with sterile adhesive film (Simport Scientific, Saint-Mathieu-de-Beloeil, QC, Canada) and incubated for 48 h at 37 °C. All combinations of bacterial strains, substances (natural phenolic compounds), and concentrations were tested in triplicate to determine both the MIC and the MBC.

The MIC was the lowest concentration of the natural substance at which no bacterial growth was noted (the well of the plate was transparent to the naked eye). *S. enterica* had higher MICs, which led to greater difficulty in visually determining microbial growth. Therefore, growth was assessed by adding 30 µL of 0.015% resazurin (Sigma Aldrich) to each well and incubating for two hours at 37 °C in the dark. Color change from blue to pink indicated microbial growth, so the lowest concentration of solution remaining blue was deemed to be the MIC.

To calculate minimum bactericidal concentrations (MBCs), 5 μL was taken from the wells and spot-plated onto TSA (Oxoid), with a selective chromogenic medium for *L. monocytogenes* (Oxoid Chromogenic *Listeria* Agar, OCLA, Oxoid Ltd.) or *Salmonella* spp. (*Salmonella* Chromogenic Agar Base, SCA, Oxoid Ltd.), as appropriate. These plates were then incubated for 24 h at 30 °C for *Listeria* spp. and 37 °C for *S. enterica*. The MBC was deemed to be the lowest concentration of the substance that achieved a total absence of bacterial growth on a given plate.

### 2.5. Statistical Analysis

Data (MIC or MBC values) were compared for statistical significance using analysis of variance techniques. Mean separations were obtained using Duncan’s multiple range test. Significant differences were established for a probability level of 5% (*p* < 0.05). Data processing was performed using the software package Statistica^®^ 8.0 (StatSoft Ltd., Tulsa, OK, USA).

## 3. Results

### 3.1. Antibiotic Resistance

The *Listeria* spp. strains tested showed resistance to one (CIP), two (CIP-RD or P-CIP), or three antibiotics (P-CIP-RD). In the case of *S. enterica*, resistance was observed for *S.* Kentucky (CIP), *S.* Typhimurium (AMP), and *S.* Typhimurium 4, [5],12:i:- (AMP) (Table 1, Tables S1 and S2).

### 3.2. Antimicrobial Activity of Ethanolic Extracts of Propolis (EEPs)

Table 2 shows compositional data for the different propolis tested. Two groups of propolis were tested: EEP1-EEP5, with high levels of CAPE and IPC (two active phenolic components that largely contribute to the pharmacological effects of propolis), and EEP6-EEP9, with low levels of such compounds. Also, the first group (EEP1–EEP5) has a greater total phenolic content than the second group (EEP6–EEP9) [36] (Tables S1 and S2).

The nine EEPs tested showed strong bacteriostatic and bactericidal activity against the 10 *Listeria* spp. strains tested. The MICs ranged from 78.00 ± 0.00 ppm to 1041.67 ± 360.84 ppm, as shown in Table 3. The MBCs ranged from 312.50 ± 0.00 ppm to 3333.33 ± 1443.38 ppm; in some cases, the MBC values were as much as eight times greater than the MIC values for most of the strains and substances studied (Table 4).

Marked differences were observed in the inhibitory effectiveness of the various EEPs against *Listeria* spp., with EEP2 having the strongest inhibitory effect and EEP6 the weakest. Striking differences were also noted from strain to strain, with ATCC 19111 being the most sensitive and STCC 937 the most resistant to most of the substances considered.

The EEPs had only slight antimicrobial effects on the *S. enterica* strains, in some cases achieving only a bacteriostatic effect, with no observed bactericidal capacity. The MIC values were 10,000 ppm or higher, depending on strain and substance, as shown in Table 5. The MBC values were >10,000 ppm in all instances, as shown in Table 6.

### 3.3. Antimicrobial Activity of Individual Phenolic Compounds

Regarding the phenolic compounds, the MIC values relative to *Listeria* spp. varied substantially. The carvacrol values ranged from 208.33 ± 72.17 ppm to 500.00 ± 0.00 ppm, whilst the eugenol values ranged from 1041.67 ± 360.84 ppm to 2500.00 ± 0.00 ppm. The thymol values were between 125.00 ± 0.00 and 288.33 ± 72.17 ppm, and for resveratrol, the values were between 125.00 ± 0.00 ppm and 500.00 ± 0.00 ppm. Finally, for trans-cinnamaldehyde, the values ranged from 166.67 ± 72.17 ppm to 333.33 ± 144.34 ppm. All the values above are shown in Table 3. The MBC values for carvacrol ranged from 500.00 ± 0.00 ppm to 666.67 ± 288.68, and the values for eugenol ranged from 2083.33 ± 721.69 ppm to 5000.00 ± 0.00 ppm. For thymol, the values were between 250.00 ± 0.00 ppm and 333.33 ± 144.34, and for resveratrol, they were between 333.33 ± 144.34 ppm and 1000.00 ± 0.00 ppm, with trans-cinnamaldehyde having a range between 250.00 ± 0.00 ppm and 500.00 ± 0.00 ppm. These details are shown in Table 4. The results highlight the strong bacteriostatic and bactericidal antimicrobial activity of thymol and trans-cinnamaldehyde. In contrast, the MIC and MBC values indicated that the highest concentrations were needed for eugenol, implying the weakest effects.

Unlike propolis, the phenolic compounds had a marked antimicrobial effect against *S. enterica*. The MIC values ranged from 250.00 ± 0.00 ppm to 500.00 ± 0.00 ppm for carvacrol and from 1250.00 ± 0.00 ppm to 2500.00 ± 0.00 ppm for eugenol. For thymol, the range was 250.00 ± 0.00 ppm to 500.00 ± 0.00 ppm, and for resveratrol, it was 500.00 ± 0.00 ppm to 1000.00 ± 0.00 ppm. The range for trans-cinnamaldehyde was 250.00 ± 0.00 ppm to 500.00 ± 0.00 ppm, as shown in Table 5. The MBC values ranged from 416.67 ± 144.34 ppm to 1000.00 ± 0.00 ppm for carvacrol, from 2500.00 ± 0.00 ppm to 5000.00 ± 0.00 ppm for eugenol, from 500.00 ± 0.00 ppm to 1000.00 ± 0.00 ppm for thymol, higher than the 1000 ± 0.00 ppm for resveratrol, and from 500.00 ± 0.00 ppm to 833.33 ± 288.68 for trans-cinnamaldehyde, as shown in Table 6. Similarly to the *Listeria* spp. results, eugenol was the least effective substance for *S. enterica*. Resveratrol had only slight bactericidal effects at the concentrations tested, with MBCs over 1000 ppm in all *S. enterica* instances (Tables S1 and S2).

## 4. Discussion

### 4.1. Geographical Relevance and Comparative Composition of Spanish Propolis

The ethanolic extracts of propolis (EEPs) analyzed in this study originate from northwestern Spain (Castilla y León) and are classified as Poplar-type propolis, based on their botanical origin and chemical profile [37]. Poplar-type propolis is the predominant chemotype in temperate Europe, typically derived from *Populus* spp. and characterized by high levels of phenolic compounds, especially flavonoids, such as pinocembrin, galangin, and chrysin, and phenolic acids, like p-coumaric acid (IPC), caffeic acid phenethyl ester (CAPE), or other derivative acid esters [37,46,47].

These samples exhibit moderate to high levels of CAPE (122.92–1690.77 ppb) and IPC (205.59–5973.60 ppb), with total polyphenol content ranging from 18.32% to 34.66% and resin content from 62.37% to 79.28%. Particularly, samples EEP 1–5 (Table 1, Section 3.3) showed consistently high values in all parameters, with polyphenol contents above 32% and IPC levels exceeding 3000 ppb, highlighting their richness in bioactive compounds.

These values are consistent with Poplar-type propolis but also suggest a distinctive regional fingerprint. Castilla y León’s flora includes extensive areas of heathland vegetation, such as *Erica* spp. (heather), *Cytisus* spp. (broom), and *Genista* spp., among others, which may contribute additional exudates to the resin collected by bees, enriching or modifying the propolis flavonoid and phenolic profile with compounds not typically found in pure Poplar-type sources. This botanical influence is consistent with the notion that propolis composition is shaped not only by taxonomy but by the resinous plant diversity accessible to bees, which varies with altitude, climate, and vegetation type [37,46,47,48].

Compared to the literature values, the chemical profile of these Spanish samples stands out. Italian Poplar-type propolis typically contains CAPE levels between 1000 and 2500 ppb and polyphenol contents between 16.7 and 24.5%, with chrysin and galangin serving as chemotaxonomic markers [49,50]. Polish propolis shows lower CAPE and IPC levels and variable polyphenol contents of 1.68–5.15% [48] and 3.03% y 14.52% [51], both under Spanish representation. Compared with other national propolis, in southern Spain, Fernández-Calderón et al. [47] described a Mediterranean-type propolis with polyphenol levels around 20.5 ± 3.4%, which are, in general, lower than those in our EEPs which have more than 22% in almost all cases (Table 2). These authors also identified a high content of unique compounds, such as vanillic acid and 3,4-DHPEA-EDA (3,4-dihydroxyphenylethanol-elenolic acid dialdehyde), a phenolic compound derived from oleuropein, characteristic of extra virgin olive oil. The identification of this compound for the first time in a propolis phenolic compound, is another sign that propolis may contain typical flora compounds of the respective geographic region, the olive in this case (*Olea Eurpaea*) a typical Mediterranean tree. In Turkey, Uzel et al. [52] reported high levels of chrysin, apigenin, and flavanones, but with wide variability in CAPE and no IPC data. In the Basque Country, Bonvehí and Gutiérrez [17] reported strong antimicrobial activity against *Salmonella enterica* (MICs 0.6–1.4 mg/mL), although no chemical composition was provided.

The consistently high phenolic and flavonoid content observed in these Spanish samples (Table 2; Section 3.3) reinforces their potential as a source of bioactive compounds, particularly for antimicrobial applications against *Listeria monocytogenes* and *Salmonella enterica*, two foodborne pathogens of major concern. While several studies have evaluated propolis activity against individual strains of these species, comprehensive assessments across multiple strains with detailed chemical correlation remain scarce. The combination of strong chemical composition, proven antimicrobial efficacy, and sustainable local sourcing positions Spanish propolis as a promising candidate for broader-spectrum applications in food safety, therapeutics, and natural preservation strategies.

Furthermore, the results support the use of CAPE and flavonoids such as chrysin and galangin as reliable chemical markers for the characterization and differentiation of propolis types, as demonstrated in both Spanish and Italian samples [37,49]. Notably, the polyphenol content of the Spanish samples—reaching up to 34.66%—is substantially higher than the ranges reported for Polish propolis (3.03–14.52%) [48,51] and Mediterranean samples (~20.5%) [49], while the presence of CAPE and IPC is more consistently quantified in the Spanish extracts. These findings reinforce the superior bioactive potential of propolis from Castilla y León.

In any case, although the Spanish propolis samples exhibit a notably higher polyphenol content compared to other European sources, including Italian propolis, this chemical richness does not always translate into superior antimicrobial activity. The results suggest that while phenolic concentration is a valuable indicator of bioactive potential, including antimicrobial activity, other factors—such as compound synergy, strain-specific susceptibility, and extract composition—also play a critical role in determining antimicrobial efficacy.

### 4.2. Antibiotic Resistance

The resistance to P, RD, and CIP observed for *L. monocytogenes* strains is worrisome because β-lactams (normally ampicillin, administered alone or in combination with gentamicin) are the first-line antibiotics for treating human listeriosis. In cases of β-lactam allergies, possible alternatives include erythromycin, vancomycin, trimethoprim/sulfamethoxazole, and fluoroquinolones [53]. Rifampicin, tetracycline, and chloramphenicol are also used to treat listeriosis [9,54]. On the other hand, some *S. enterica* strains showed resistance to AMP (penicillin) or CIP (fluoroquinolone). Both antibiotics have long been considered drugs of choice to treat salmonellosis when necessary [2,14,15,43].

Although the overall resistance levels were not extreme, with low levels in comparison with other works [18], the strains tested include reference and epidemiologically relevant isolates, making them representative of clinically significant profiles. This reinforces the importance of evaluating alternative antimicrobial strategies—not only for resistant strains but also for sensitive ones—given the potential for emerging resistance and the need for preventive approaches.

Natural compounds such as propolis and plant-derived terpenes have shown promising bioactive potential, with several studies demonstrating their efficacy against *Listeria monocytogenes* and *Salmonella enterica*, including synergistic effects with conventional antibiotics [18,19,20,22,31]. Their relevance extends beyond clinical applications, offering innovative solutions for the food industry, particularly in the development of natural disinfectants and preservation strategies.

### 4.3. Natural Phenolic Compound Effectiveness

The antimicrobial effects of nine EEPs and five phenolic compounds upon 10 strains of *Listeria* spp. and 10 strains of *S. enterica* were determined in this research work. These bacteria are frequently involved in sporadic foodborne disease cases and outbreaks in many EU countries, with high fatality (*Listeria* spp.) and incidence (*Salmonella*) rates, respectively [3].

Numerous studies have demonstrated that propolis is active against various microorganisms [17,18,27,46,47,48,49,51]. Moreover, the substances identified in propolis are familiar constituents of food, food additives, and/or Generally Recognized as Safe (GRAS) substances [25,55]. The components of propolis with the strongest antimicrobial effects are terpenes, flavonoids, and phenolic acids, several of them belonging to the GRAS group. These substances act simultaneously on various target parts of bacteria, so propolis poses a threat to bacterial survival in different ways at the same time. This multitarget mode of action reduces the likelihood of resistance emergence [19,22,26].

The EEPs tested showed marked antimicrobial activity against all the *Listeria* spp. strains. However, they had little impact on *S. enterica*. Most of the published works indicated that Gram-positive bacteria are more sensitive to propolis than Gram-negative bacteria [18,27,44,45,47,48,51]. This lower sensitivity of Gram-negative bacteria to propolis is due to their external structure, as their external membranes can render access by substances to the interior of the cell difficult or even impossible, especially for hydrophilic or large molecules, as it is the case of many phenolic compounds [18,22,27,31,48,49,51].

The MICs recorded in this research for *Listeria* spp., between 78 and 1250 ppm, were like those observed by other authors for Gram-positive bacteria, as indicated below (Table 3). Al-Ani et al. [18] noted MIC values between 40 and 1200 ppm for strains of *Bacillus subtilis*, *Streptococcus* spp., and *Staphylococcus aureus*. Grecka et al. [51] recorded MIC values of 128 ppm in the case of *S. epidermidis* and *S. aureus* of clinical origin. In contrast, other reports observed poor efficacy of propolis against *L. monocytogenes*. Thus, Pobiega et al. [48] studied the effects of different types of propolis from Poland on various pathogenic microorganisms and noted high MIC and MBC values against *L. monocytogenes* in the range 4000 and 16,000 ppm. The different results observed in the various research works may be attributable to differences between the strains, types of propolis, phenolic composition, and conditions involved in these investigations.

Caffeic acid phenethyl ester (CAPE) is one of the most abundant components in propolis samples of different varieties and geographical areas, especially in Europe, and, furthermore, it is one of the phenolic compounds with greater biological activity [37,46,47,51]. No differences were observed in the antimicrobial activity of propolis based on their CAPE or isoprenyl caffeate (IPC) content, not finding a relationship/answer based on the content of said compound. Therefore, the antimicrobial efficacy of propolis in this study is most likely due to other phenolic components, since it may contain over 300 different chemical constituents, the majority of which are phytochemicals, given its mainly plant origin.

The antimicrobial effect of EEPs against *L. monocytogenes* strains resistant to antibiotics used to treat human listeriosis (P, RD, CIP) suggests the usefulness of propolis in clinical practice. However, additional investigations should be carried out to confirm this suggestion.

The limited sensitivity of Gram-negative bacteria to propolis observed in this study is consistent with the findings of Al-Ani et al. [18], who recorded MIC values above 2500 ppm and MBC values greater than 5000 ppm in different groups of bacteria (*Escherichia coli*, *Pseudomonas aeruginosa*, *Yersinia enterocolitica*, *Klebsiella pneumoniae*, and *Salmonella*). Pobiega et al. [48] also found high MIC and MBC values for *S.* Enteritidis, ranging from 8000 to 32,000 ppm. In contrast, considering propolis from northern Spain, Bonvehí et al. [17] noted MIC values between 600 and 1200 ppm for *S. enterica*. These results suggest that propolis composition, the cellular envelope structure and composition in Gram-positive and Gram-negative bacteria, and intraspecific variability in the strains influence the results obtained. Additionally, the predominance of flavonoids and phenolic acids in propolis, as is our case, may contribute to its limited efficacy against *Salmonella*. These compounds generally have larger molecular structures and higher polarity compared to terpenes (highly lipophilic), which hinders their ability to cross the outer membrane of Gram-negative bacteria [19,21,27,48].

The five isolate phenolic compounds tested are safe plant-derived molecules. Thymol and carvacrol (whether chemical or natural) are safe according to the European Commission and have received GRAS (Generally Recognized as Safe) status from the US Food and Drug Administration (FDA). These compounds are classified as flavoring agents; hence, they meet regulatory requirements as additives for food preservation [25]. Eugenol has also received GRAS status from the FDA as a direct human food ingredient. Similarly, this molecule has been approved by the European Commission as an active substance with no maximum residue level (MRL) required for several food applications [55]. Resveratrol is a GRAS natural product with a history of human consumption [30,53,55]. In the European Union, it is considered a safe novel food [56]. Lastly, trans-cinnamaldehyde is considered a GRAS substance by the FDA [57] and has received a positive safety evaluation by the European Food Safety Authority (EFSA) [57].

The individual phenolic substances tested, mainly terpenes, had a marked antimicrobial effect on both *Listeria* spp. and *S. enterica*. These results agree with those observed by other authors. Liu et al. [33] studied the antimicrobial effect of eugenol, thymol, and trans-cinnamaldehyde on both groups of bacteria and obtained MIC and MBC values similar to those in the present work; eugenol was the least effective substance, as observed in this study. However, unlike in this study, where thymol had MICs between 250 and 500 ppm for all strains, without differences between species, the above-mentioned authors noted greater effectiveness of this compound against *S.* Typhimurium (250 ppm) than against *L. monocytogenes* (1024 ppm). Inter-strain variability could be responsible, at least partially, for the differences in these results.

Miladi et al. [32] analyzed the effects of carvacrol, eugenol, and thymol on various *S.* Typhimurium strains and noted values ranging from 64 to 1024 ppm, a finding in accord with the results presented here. As in the present investigation, these authors observed higher MIC and MBC values for eugenol than for the remaining compounds, confirming that the monoterpenes carvacrol, thymol, and trans-cinnamaldehyde are somewhat more effective than eugenol. This substance (i.e., eugenol) has a greater molecular size, so it is difficult for it to enter cells. Mith et al. [34] analyzed the antimicrobial effectiveness of five common terpenoids that are abundant in essential oils (linalool, carvacrol, eugenol, thymol, and trans-cinnamaldehyde) against *L. monocytogenes*, *S.* Typhimurium, and *E. coli* 0157:H7 strains. They observed that carvacrol and trans-cinnamaldehyde had lower MIC and MBC values than the other compounds against all strains. By contrast, these authors observed the low antimicrobial effectiveness of eugenol, which is congruent with the results reported here.

Resveratrol is a substance of plant origin belonging to the stilbene group. This compound has a defensive function; it is active against various pathogenic microorganisms, its effect being principally bacteriostatic [30,36,39]. Ferreira and Domingues [36] noted MICs for resveratrol that were very similar to those in this study for *L. monocytogenes*, with values around 200 ppm, demonstrating that this substance is quite an effective treatment for controlling that pathogen. The antimicrobial impact of resveratrol in the present study for *S. enterica* was very slight, with MBC values of 1000 ppm or more, like those recorded by Paulo et al. [30] and Tegos et al. [29]. These authors observed values ≥400 to 500 ppm, which highlights the greater resistance of Gram-negative bacteria, such as *S. enterica*, to this substance [42]. In general, although resveratrol demonstrated antimicrobial activity, it is a stilbene-type compound rather than a terpene. Its rigid, polyphenolic structure may similarly restrict membrane permeability, thereby limiting its overall efficacy against Gram-negative bacteria. In fact, Karamese and Dicle [58] reported that resveratrol required relatively high concentrations to inhibit Gram-negative bacteria and biofilm formation, further suggesting its lower potency compared to terpenes. Furthermore, it should be noted that the limited solubility of resveratrol in water causes problems in its extraction and application.

In general, although EEPs are rich in phenolic compounds, particularly flavonoids and phenolic acids [17], their antimicrobial efficacy against *S. enterica* appears limited (10,000 ppm or more). As previously mentioned, this may be due to the structural characteristics of these compounds, which often include larger molecular sizes and higher polarity, reducing their ability to penetrate the outer membrane of Gram-negative bacteria [19,21]. In contrast, terpenes such as thymol, carvacrol, and trans-cinnamaldehyde are small, lipophilic molecules that can easily integrate into bacterial membranes, disrupt their integrity, and cause intracellular content leakage [22,27]. Moreover, Pobiega et al. [48] reported high MIC and MBC values for Polish propolis extracts against *S.* Enteritidis, supporting the idea that the phenolic profile of propolis may not be optimal for targeting Gram-negative pathogens. This is consistent with the findings of Adanero-Jorge et al. [37], who characterized propolis samples from Castilla y León and reported a composition dominated by flavonoids and phenolic acids, with low or negligible terpene content, which may explain the limited activity observed against *Salmonella* in this study.

Therefore, the apparent contradiction between the presence of phenolic compounds in EEPs and their limited efficacy against *S. enterica* can be explained by the complexity of natural extracts [18,19,22,27,31]. Unlike purified compounds, EEPs contain a mixture of constituents whose interactions may reduce bioavailability or lead to antagonistic effects. Additionally, the concentration of active phenolics, such as terpenes, in EEPs may be insufficient or too low to exert a strong antimicrobial effect, especially against resilient Gram-negative species, or due to the higher molecular size of flavonoids and phenolic acids [21,27,46,48,49,51]. Notably, phenolic compounds may be more effective against *L. monocytogenes*, a Gram-positive bacterium lacking an outer membrane, which facilitates the penetration of polar molecules and enhances their antimicrobial action [18,48,52,59]. All these elements address the specific limitations of flavonoids and phenolic acids (polar phenols) in Gram-negative systems compared with terpenes (lipophilic and less/non-polar phenols), which are critical to understanding the reduced efficacy of EEPs against Gram-negative strains in this context.

Collectively, these findings underscore the superior performance of individual terpenes against *Salmonella*, not only due to their favorable physicochemical properties and mechanisms of action—such as membrane disruption and enhanced permeability—but also because of their broader spectrum of activity. They are effective against *both Listeria monocytogenes* and *Salmonella enterica*, including all tested strains, making them more suitable candidates for controlling Gram-negative pathogens.

## 5. Conclusions

The propolis extracts and phenolic compounds tested demonstrated antimicrobial activity against both *Listeria* spp. and *S. enterica* strains that show resistance and non-resistance to antibiotics. This implies they could be an alternative to or complement conventional antimicrobial treatments based on antibiotics and biocides. Propolis is most effective against Gram-positive bacteria. In contrast, phenolic compounds showed marked antimicrobial activity against both bacterial groups and strains (*Listeria* spp. and *S. enterica*). Nevertheless, major differences were observed between these substances, with thymol having the greatest antimicrobial effect and eugenol the least. However, it should be noted that these results were obtained in laboratory trials with *in vitro* experiments, and further studies are required to identify the mechanism of action of these natural substances and to determine their antimicrobial effectivity in field conditions. The effectiveness of EEPs and phenolic compounds in preventing biofilm formation or eradicating such structures should also be established. In addition, it would be desirable to investigate combinations of such compounds with other antimicrobials (antibiotics or biocides) to uncover the type of effect obtained.

## Figures and Tables

**Table 1:** Antibiotic resistance patterns of the bacteria tested.

Strain	Pattern of Antibiotic Resistance
*Listeria* spp.	
ATCC 19111	CIP
ATCC 19112	CIP
ATCC 19114	CIP-RD
ATCC 19117	P-CIP-RD
ATCC 13932	P-CIP-RD
STCC 936	CIP
STCC 937	P-CIP-RD
STCC 938	P-CIP
C6	P-CIP
ATCC 19119	CIP
*Salmonella enterica*	
*S.* Agona	-
*S.* Enteritidis	-
*S.* Hadar	-
*S.* Kentucky	CIP
*S.* Infantis	.
*S.* London	-
*S.* Newport	-
*S.* Typhimurium	AMP
*S.* Typhimurium 1, 4, [5], 12:i:-	AMP
*S.* Virchow	-

ATCC, American Type Culture Collection; STCC, Spanish Type Culture Collection; C6, isolate from poultry. Nine strains of *L. monocytogenes* were tested: ATCC 19111 (serotype 1/2b), ATCC 19112 (serotype 1/2b), ATCC 19114 (serotype 4a), ATCC 19117 (serotype 4d), ATCC 13932 (serotype 4b), STCC 936 (serotype 1/2b), STCC 937 (serotype 3b), STCC 938 (serotype 3c), and C6 (strain isolated from pork). Additionally, *L. ivanovii* ATCC 19119 was investigated. The following discs were used for *Listeria* spp.: AMP (ampicillin, 10 μg), AMC (amoxicillin/clavulanic acid, 30 μg), P (penicillin, 10 units), SXT (trimethoprim/sulfamethoxazole, 25 μg), CN (gentamicin, 10 μg), E, (erythromycin, 15 μg), CIP (ciprofloxacin, 5 μg), TE (tetracycline, 30 μg), VA (vancomycin 30 μg), and RD (rifampicin, 5 μg). The antibiotics tested for *S. enterica* were AMP, AMC, CRO (ceftriaxone, 30 μg), AZM (azithromycin, 15 μg), SXT, CIP, C (chloramphenicol, 30 μg), MEM (meropenem, 10 μg), IMP (imipenem, 10 μg), and FOS (fosfomycin, 200 mg).

**Table 2:** Chemical composition of the propolis tested.

Propolis	CAPE (ppb)	IPC (ppb)	Resins (%)	Total Polyphenols (%)
EEP 1	1302.14	3653.47	79.28	32.22
EEP 2	1408.14	3341.46	72.66	33.30
EEP 3	1539.82	3477.53	78.65	32.86
EEP 4	1690.77	5645.67	72.56	33.68
EEP 5	1198.98	5973.60	75.47	34.66
EEP 6	228.84	205.59	71.64	22.43
EEP 7	370.29	382.80	78.09	26.30
EEP8	288.82	224.55	66.81	22.05
EEP 9	122.92	535.27	62.37	18.32

CAPE, caffeic acid phenethyl ester; IPC, isoprenyl caffeate; ppb, parts per billion (ng/mL).

**Table 3:** Minimum inhibitory concentrations (MICs, ppm) for nine ethanolic extracts of propolis (EEPs) and five phenolic compounds with respect to ten *Listeria* spp. strains.

Antimicrobial Compound	*Listeria* Strains									
	*Listeria monocytogenes*									*Listeria ivanovii*
	ATCC ^1^ 19111	ATCC 19112	ATCC 19114	ATCC 19117	ATCC 13932	STCC ^2^ 936	STCC 937	STCC 938	C6 ^3^	ATCC 19119
**EEP ^4^ 1**	312.50 ± 0.00 ^a^_c_	417.67 ± 180.42 ^ab^_bc_	520.83 ± 180.42 ^ab^_de_	520.83 ± 180.42 ^ab^_d_	312.50 ± 0.00 ^a^_c_	520.83 ± 180.42 ^ab^_b_	625.00 ± 0.00 ^b^_bcd_	520.83 ± 180.42 ^ab^_c_	625.00 ± 0.00 ^b^_b_	416.67 ± 180.42 ^ab^_b_
**EEP2**	78.00 ± 0.00 ^a^_a_	182.29 ± 119.34 ^b^_ab_	156.25 ± 0.00 ^b^_a_	312.50 ± 0.00 ^c^_bc_	312.50 ± 0.00 ^c^_c_	312.50 ± 0.00 ^c^_a_	625.00 ± 0.00 ^d^_bcd_	312.50 ± 0.00 ^c^_ab_	156.25 ± 0.00 ^b^_a_	78.00 ± 0.00 ^a^_a_
**EEP3**	208.33 ± 90.21 ^a^_abc_	312.50 ± 0.00 ^ab^_abc_	468.75 ± 270.63 ^bc^_cde_	312.50 ± 0.00 ^ab^_bc_	312.50 ± 0.00 ^ab^_c_	312.50 ± 0.00 ^ab^_a_	625.00 ± 0.00 ^c^_bcd_	312.50 ± 0.80 ^ab^_ab_	312.50 ± 0.00 ^ab^_ab_	260.42 ± 90.21 ^a^_ab_
**EEP4**	130.17 ± 45.18 ^a^_ab_	312.50 ± 0.00 ^b^_abc_	156.25 ± 0.00 ^a^_a_	416.67 ± 180.42 ^b^_c_	312.50 ± 0.00 ^b^_c_	312.50 ± 0.00 ^b^_a_	625.00 ± 0.00 ^c^_bcd_	416.67 ± 180.42 ^b^_bc_	156.25 ± 0.00 ^a^_a_	78.00 ± 0.00 ^a^_a_
**EEP5**	260.42 ± 90.21 ^ab^_bc_	312.50 ± 0.00 ^b^_abc_	312.50 ± 0.00 ^b^_abcd_	312.50 ± 0.00 ^b^_bc_	312.50 ± 0.00 ^b^_c_	312.50 ± 0.00 ^b^_a_	625.00 ± 0.00 ^c^_bcd_	312.50 ± 0.00 ^b^_ab_	312.50 ± 0.00 ^b^_ab_	208.33 ± 90.21 ^a^_ab_
**EEP6**	260.42 ± 90.21 ^a^_bc_	1041.67 ± 360.84 ^c^_d_	468.75 ± 270.63 ^ab^_cde_	312.50 ± 0.00 ^a^_bc_	312.50 ± 0.00 ^a^_c_	260.42 ± 90.21 ^a^_a_	625.00 ± 0.00 ^b^_bcd_	312.50 ± 0.00 ^a^_ab_	416.67 ± 180.42 ^ab^_ab_	260.42 ± 90.21 ^a^_ab_
**EEP7**	156.17 ± 135.39 ^a^_ab_	312.50 ± 0.00 ^a^_abc_	625.00 ± 0.00 ^b^_e_	260.42 ± 90.21 ^a^_ab_	312.50 ± 0.00 ^a^_c_	260.42 ± 90.21 ^a^_a_	625.00 ± 0.00 ^b^_bcd_	260.42 ± 90.21 ^a^_ab_	208.33 ± 90.21 ^a^_a_	234.33 ±135.39 ^a^_ab_
**EEP8**	156.17 ± 135.39 ^a^_ab_	312.50 ± 0.00 ^ab^_abc_	468.75 ± 270.63 ^bc^_cde_	156.25 ± 0.00 ^a^_a_	208.33 ± 90.21 ^a^_a_	208.33 ± 90.21 ^a^_a_	625.00 ± 0.00 ^c^_bcd_	312.50 ± 0.00 ^ab^_ab_	156.25 ± 0.00 ^a^_a_	312.50 ± 0.00 ^ab^_ab_
**EEP9**	130.17 ± 45.18 ^a^_ab_	520.83 ± 180.42 ^b^_c_	156.25 ± 0.00 ^a^_a_	312.50 ± 0.00 ^ab^_bc_	312.50 ± 0.00 ^ab^_c_	520.83 ± 180.42 ^b^_b_	833.33 ± 360.84 ^c^_d_	416.67 ± 180.42 ^ab^_bc_	312.50 ± 0.00 ^ab^_ab_	156.25 ± 0.00 ^a^_a_
**Carvacrol**	208.33 ± 72.17 ^a^_abc_	250.00 ± 0.00 ^a^_ab_	250.00 ± 0.00 ^a^_abc_	250.00 ± 0.00 ^a^_ab_	250.00 ± 0.00 ^a^_b_	250.00 ± 0.00 ^a^_a_	250.00 ± 0.00 ^a^_abc_	250.00 ± 0.00 ^a^_ab_	500.00 ± 0.00 ^b^_ab_	208.33 ± 72.17 ^a^_ab_
**Eugenol**	1250.00 ± 0.00 ^a^_d_	1250.00 ± 0.00 ^a^_e_	1250.00 ± 0.00 ^a^_f_	2500.00 ± 0.00 ^c^_e_	1250.00 ± 0.00 ^a^_d_	1250 ± 0.00 ^a^_c_	1666.67 ± 721.69 ^ab^_e_	2500.00 ± 0.00 ^c^_d_	2083.33 ± 721.69 ^bc^_c_	1041.67 ± 360.84 ^a^_c_
**Thymol**	125.00 ± 0.00 ^a^_ab_	250.00 ± 0.00 ^b^_ab_	208.33 ± 72.17 ^b^_ab_	250.00 ± 0.00 ^b^_ab_	250.00 ± 0.00 ^b^_b_	288.33 ± 72.17 ^b^_a_	125.00 ± 0.00 ^a^_a_	208.33 ± 72.17 ^b^_a_	250.00 ± 0.00 ^b^_ab_	125.00 ± 0.00 ^a^_a_
**Resveratrol**	125.00 ± 0.00 ^a^_ab_	250.00 ± 0.00 ^c^_ab_	250.00 ± 0.00 ^c^_abc_	250.00 ± 0.00 ^c^_ab_	250.00 ± 0.00 ^c^_b_	250.00 ± 0.00 ^c^_a_	250.00 ± 0.00 ^c^_abc_	250.00 ± 0.00 ^c^_ab_	500.00 ± 0.00 ^d^_ab_	166.67 ± 72.17 ^b^_a_
***Trans*-** ** cinnamaldehyde **	250.00 ± 0.00 ^ab^_bc_	166.67 ± 72.17 ^a^_a_	250.00 ± 0.00 ^ab^_abc_	250.00 ± 0.00 ^ab^_ab_	250.00 ± 0.00 ^ab^_b_	250.00 ± 0.00 ^ab^_a_	250.00 ± 0.00 ^ab^_abc_	250.00 ± 0.00 ^ab^_ab_	333.33 ± 144.34 ^b^_ab_	250.00 ± 0.00 ^ab^_ab_
***Solvent Control (7% Ethanol)***	Growth									

^1^, American Type Culture Collection; ^2^, Spanish Type Culture Collection; ^3^, C6, isolate from pork; ^4^, ethanolic extracts of propolis. Data (mean ± STD; n = 3) in the same row (*Listeria* spp. strain comparison) with no letters in common (superscript) are significantly different (*p* < 0.05). Means in the same column (antimicrobial compound comparison) with no letters in common (subscript) are significantly different (*p* < 0.05).

**Table 4:** Minimum bactericidal concentrations (MBCs, ppm) for nine ethanolic extracts of propolis (EEPs) and five phenolic compounds with respect to ten *Listeria* spp. strains.

Antimicrobial Compound	*Listeria* Strains									
	*Listeria monocytogenes*									*Listeria ivanovii*
	ATCC ^1^ 19111	ATCC 19112	ATCC 19114	ATCC 19117	ATCC 13932	STCC ^2^ 936	STCC 937	STCC 938	C6 ^3^	ATCC 19119
**EEP1**	2083.33 ± 721.69 ^ab^_e_	2500.00 ± 0.00 ^b^_g_	2083.33 ± 2525.91 ^ab^_bc_	1250.00 ± 0.00 ^ab^_c_	625.00 ± 0.00 ^a^_ab_	625.00 ± 0.00 ^a^_abc_	2500.00 ± 0.00 ^b^_d_	2083.33 ± 721.69 ^ab^_bc_	5000.00 ± 0.00 ^c^_e_	520.83 ± 180.42 ^a^_ab_
**EEP2**	625.00 ± 0.00 ^ab^_abc_	2083.33 ± 721.69 ^c^_fg_	833.33 ± 360.84 ^ab^_ab_	625.00 ± 0.00 ^ab^_abc_	1458.33 ± 954.70 ^bc^_c_	625.00 ± 0.00 ^ab^_abc_	1250.00 ± 0.00 ^abc^_c_	1458.33 ± 954.70 ^bc^_abc_	312.50 ± 0.00 ^a^_a_	625.00 ± 0.00 ^ab^_ab_
**EEP3**	937.50 ± 541.27 ^a^_bcd_	1250.00 ± 0.00 ^ab^_de_	625.00 ± 0.00 ^a^_ab_	625.00 ± 0.00 ^a^_abc_	1250.00 ± 0.00 ^ab^_bc_	1458.33 ± 954.70 ^ab^_d_	1250.00 ± 0.00 ^ab^_c_	2500.00 ± 2165.06 ^b^_c_	2500.00 ± 0.00 ^b^_c_	729.17 ± 477.35 ^a^_ab_
**EEP4**	1250.00 ± 0.00 ^d^_d_	1041.67 ± 360.84 ^cd^_cd_	625.00 ± 0.00 ^ab^_ab_	833.33 ± 360.84 ^bc^_abc_	1250.00 ± 0.00 ^d^_bc_	1041.67 ± 360.84 ^cd^_bcd_	1250.00 ± 0.00 ^d^_c_	625.00 ± 0.00 ^ab^_ab_	312.50 ± 0.00 ^a^_a_	625.00 ± 0.00 ^ab^_ab_
**EEP5**	625.00 ± 0.00 ^a^_abc_	1250.00 ± 0.00 ^b^_de_	833.33 ± 360.84 ^ab^_ab_	625.00 ± 0.00 ^a^_abc_	833.33 ± 360.84 ^ab^_abc_	1041.67 ± 360.84 ^ab^_bcd_	1041.67 ± 360.84 ^ab^_bc_	625.00 ± 0.00 ^a^_ab_	833.33 ± 360.84 ^ab^_ab_	625.00 ± 0.00 ^a^_ab_
**EEP6**	1041.67 ± 360.84 ^a^_cd_	1250.00 ± 0.00 ^a^_de_	520.83 ± 180.42 ^a^_a_	1041.67 ± 360.84 ^a^_bc_	1250.00 ± 0.00 ^a^_bc_	312.50 ± 0.00 ^a^_a_	1250.00 ± 0.00 ^a^_c_	1041.67 ± 360.84 ^a^_abc_	3333.33 ± 1443.38 ^b^_d_	1041.67 ± 36.84 ^a^_b_
**EEP7**	625.00 ± 541.27 ^a^_abc_	833.33 ± 360.84 ^ab^_bcd_	1250.00 ± 1082.53 ^ab^_abc_	1875.00 ± 1082.53 ^ab^_d_	625.00 ± 0.00 ^a^_ab_	625.00 ± 541.27 ^a^_abc_	2083.33 ± 721.69 ^b^_d_	1250.00 ± 1082.53 ^ab^_abc_	833.33 ± 360.84 ^ab^_ab_	625.00 ± 541.27 ^a^_ab_
**EEP8**	625.00 ± 0.00 ^ab^_abc_	1666.67 ± 721.69 ^c^_ef_	1250.00 ± 0.00 ^bc^_abc_	1250.00 ± 0.00 ^bc^_c_	312.50 ± 0.00 ^a^_a_	833.33 ± 360.84 ^ab^_abcd_	1250.00 ± 0.00 ^bc^_c_	1250.00 ± 1082.53 ^bc^_abc_	1250.00 ± 0.00 ^bc^_b_	625.00 ± 0.00 ^ab^_ab_
**EEP9**	833.33 ± 360.84 ^a^_abcd_	2500.00 ± 0.00 ^b^_g_	625.00 ± 0.00 ^a^_ab_	833.33 ± 360.84 ^a^_abc_	1250.00 ± 1082.53 ^a^_bc_	1250.00 ± 0.00 ^a^_cd_	2083.00 ± 721.69 ^b^_d_	416.67 ± 180.42 ^a^_a_	625.00 ± 0.00 ^a^_ab_	625.00 ± 0.00 ^a^_ab_
**Carvacrol**	666.67 ± 288.68 ^a^_abcd_	500.00 ± 0.00 ^a^_ab_	666.67 ± 288.68 ^a^_ab_	500.00 ± 0.00 ^a^_ab_	666.67 ± 288.68 ^a^_ab_	666.67 ± 288.68 ^a^_abc_	666.67 ± 288.68 ^a^_ab_	500.00 ± 0.00 ^a^_a_	500.00 ± 0.00 ^a^_ab_	583.33 ± 381.88 ^a^_ab_
**Eugenol**	2500.00 ± 0.00 ^a^_e_	2500.00 ± 0.00 ^a^_g_	2500.00 ± 0.00 ^a^_c_	5000.00 ± 0.00 ^b^_e_	2500.00 ± 0.00 ^a^_d_	2500.00 ± 0.00 ^a^_e_	2500.00 ± 0.00 ^a^_d_	5000.00 ± 0.00 ^b^_d_	5000.00 ± 0.00 ^b^_e_	2083.33 ± 721.69 ^a^_c_
**Thymol**	250.00 ± 0.00 ^a^_a_	250.00 ± 0.00 ^a^_a_	250.00 ± 0.00 ^a^_a_	250.00 ± 0.00 ^a^_a_	250.00 ± 0.00 ^a^_a_	333.33 ± 144.34 ^a^_a_	250.00 ± 0.00 ^a^_a_	250.00 ± 0.00 ^a^_a_	333.33 ± 144.34 ^a^_a_	250.00 ± 0.00 ^a^_a_
**Resveratrol**	333.33 ± 144.34 ^a^_ab_	500.00 ± 0.00 ^ab^_ab_	833.33 ± 288.68 ^bc^_ab_	667.67 ± 288.68 ^abc^_abc_	1000.00 ± 0.00 ^c^_abc_	833.33 ± 288.68 ^bc^_abcd_	666.67 ± 288.68 ^abc^_ab_	667.67 ± 288.68 ^abc^_ab_	1000.00 ± 0.00 ^c^_ab_	333.33 ± 144.34 ^a^_a_
***Trans*-** ** cinnamaldehyde **	500.00 ± 0.00 ^b^_abc_	250.00 ± 0.00 ^a^_a_	500.00 ± 0.00 ^b^_a_	250.00 ± 0.00 ^a^_a_	250.00 ± 0.00 ^a^_a_	500.00 ± 0.00 ^b^_ab_	250.00 ± 0.00 ^a^_a_	250.00 ± 0.00 ^a^_a_	333.33 ± 144.34 ^a^_a_	250.00 ± 0.00 ^a^_a_

^1^, American Type Culture Collection; ^2^, Spanish Type Culture Collection; ^3^, C6, isolate from pork. Data (mean ± STD; n = 3) in the same row (*Listeria* spp. strain comparison) with no letters in common (superscript) are significantly different (*p* < 0.05). Means in the same column (antimicrobial compound comparison) with no letters in common (subscript) are significantly different (*p* < 0.05).

**Table 5:** Minimum inhibitory concentration (MICs, ppm) for nine ethanolic extract of propolis (EEP) and five phenolic compounds with respect to 10 *Salmonella enterica* strains.

Antimicrobial Compound	*Salmonella enterica* Strains									
	*S.* Agona	*S.* Enteritidis	*S.* Hadar	*S.* Infantis	*S.* Kentucky	*S.* London	*S.* Newport	*S.* Typhimurium	*S.* Typhimurium *monophasic* Variant 1,4,(5),12:i:-	*S.* Virchow
**EEP1**	>10,000.00	10,000.00 ± 0.00	>10,000.00	>10,000.00	10,000.00 ± 0.00	10,000.00 ± 0.00	>10,000.00	>10,000.00	>10,000.00	>10,000.00
**EEP2**	10,000.00 ± 0.00	10,000.00 ± 0.00	10,000.00 ± 0.00	10,000.00 ± 0.00	>10,000.00	>10,000.00	10,000.00 ± 0.00	10,000.00 ± 0.00	10,000.00 ± 0.00	10,000.00 ± 0.00
**EEP3**	>10,000.00	>10,000.00	>10,000.00	>10,000.00	>10,000.00	>10,000.00	>10,000.00	>10,000.00	>10,000.00	>10,000.00
**EEP4**	10,000.00 ± 0.00	>10,000.00	10,000.00 ± 0.00	>10,000.00	>10,000.00	>10,000.00	>10,000.00	>10,000.00	10,000.00 ± 0.00	>10,000.00
**EEP5**	>10,000.00	>10,000.00	>10,000.00	10,000.00 ± 0.00	>10,000.00	>10,000.00	>10,000.00	>10,000.00	>10,000.00	>10,000.00
**EEP6**	>10,000.00	10,000.00 ± 0.00	>10,000.00	10,000.00 ± 0.00	10,000.00 ± 0.00	10,000.00± 0.00	10,000.00 ± 0.00	10,000.00 ± 0.00	10,000.00 ± 0.00	10,000.00
**EEP7**	>10,000.00	>10,000.00	>10,000.00	>10,000.00	>10,000.00	>10,000.00	>10,000.00	>10,000.00	>10,000.00	>10,000.00
**EEP8**	>10,000.00	>10,000.00	>10,000.00	10,000.00 ± 0.00	10,000.00 ± 0.00	>10,000.00	>10,000.00	>10,000.00	>10,000.00	>10,000.00
**EEP9**	10,000.00 ± 0.00	>10,000.00	>10,000.00	>10,000.00	>10,000.00	>10,000.00	10,000.00 ± 0.00	>10,000.00	10,000.00 ± 0.00	10,000.00 ± 0.00
**Carvacrol**	250.00 ± 0.00	250.00 ± 0.00	500.00 ± 0.00	250.00 ± 0.00	500.00 ± 0.00	250.00 ± 0.00	250.00 ± 0.00	500.00 ± 0.00	250.00 ± 0.00	250.00 ± 0.00
**Eugenol**	1250.00 ± 0.00	2500.00 ± 0.00	2500.00 ± 0.00	1667.67 ± 721.69	2500.00 ± 0.00	1250.00 ± 0.00	1250.00 ± 0.00	2500.00 ± 0.00	1250.00 ± 0.00	2500.00 ± 0.00
**Thymol**	250.00 ± 0.00	250.00 ± 0.00	500.00 ± 0.00	250.00 ± 0.00	250.00 ± 0.00	250.00 ± 0.00	250.00 ± 0.00	500.00 ± 0.00	250.00 ± 0.00	250.00 ± 0.00
**Resveratrol**	500.00 ± 0.00	1000.00 ± 0.00	500.00 ± 0.00	1000.00 ± 0.00	1000.00 ± 0.00	500.00 ± 0.00	500.00 ± 0.00	1000.00 ± 0.00	500.00 ± 0.00	1000.00 ± 0.00
***Trans*-** ** cinnamaldehyde **	250.00 ± 0.00	250.00 ± 0.00	500.00 ± 0.00	500.00 ± 0.00	250.00 ± 0.00	500.00 ± 0.00	250.00 ± 0.00	500.00 ± 0.00	500.00 ± 0.00	500.00 ± 0.00

*Salmonella* strains were isolated from poultry.

**Table 6:** Minimum bactericidal concentrations (MBCs, ppm) for nine ethanolic extracts of propolis (EEPs) and five phenolic compounds with respect to ten *Salmonella enterica* strains.

Antimicrobial Compound	*Salmonella enterica* Strains									
	*S.* Agona	*S.* Enteritidis	*S.* Hadar	*S.* Infantis	*S.* Kentucky	*S.* London	*S.* Newport	*S.* Typhimurium	*S.* Typhimurium *monophasic* Variant 1,4,(5),12:i:-	*S.* Virchow
**EEP1**	>10,000.00	>10,000.00	>10,000.00	>10,000.00	>10,000.00	>10,000.00	>10,000.00	>10,000.00	>10,000.00	>10,000.00
**EEP2**	>10,000.00	>10,000.00	>10,000.00	>10,000.00	>10,000.00	>10,000.00	>10,000.00	>10,000.00	>10,000.00	>10,000.00
**EEP3**	>10,000.00	>10,000.00	>10,000.00	>10,000.00	>10,000.00	>10,000.00	>10,000.00	>10,000.00	>10,000.00	>10,000.00
**EEP4**	>10,000.00	>10,000.00	>10,000.00	>10,000.00	>10,000.00	>10,000.00	>10,000.00	>10,000.00	>10,000.00	>10,000.00
**EEP5**	>10,000.00	>10,000.00	>10,000.00	>10,000.00	>10,000.00	>10,000.00	>10,000.00	>10,000.00	>10,000.00	>10,000.00
**EEP6**	>10,000.00	>10,000.00	>10,000.00	>10,000.00	>10,000.00	>10,000.00	>10,000.00	>10,000.00	>10,000.00	>10,000.00
**EEP7**	>10,000.00	>10,000.00	>10,000.00	>10,000.00	>10,000.00	>10,000.00	>10,000.00	>10,000.00	>10,000.00	>10,000.00
**EEP8**	>10,000.00	>10,000.00	>10,000.00	>10,000.00	>10,000.00	>10,000.00	>10,000.00	>10,000.00	>10,000.00	>10,000.00
**EEP9**	>10,000.00	>10,000.00	>10,000.00	>10,000.00	>10,000.00	>10,000.00	>10,000.00	>10,000.00	>10,000.00	>10,000.00
**Carvacrol**	416.67 ± 144.34	1000.00 ± 0.00	1000.00 ± 0.00	500.00 ± 0.00	1000.00 ± 0.00	500.00 ± 0.00	500.00 ± 0.00	416.67 ± 144.34	416.67 ± 144.34	666.67 ± 288.68
**Eugenol**	2500.00 ± 0.00	5000.00 ± 0.00	2500.00 ± 0.00	2500.00 ± 0.00	5000.00 ± 0.00	2500.00 ± 0.00	2500.00 ± 0.00	5000.00 ± 0.00	2500.00 ± 0.00	5000.00 ± 0.00
**Thymol**	833.33 ± 288.68	1000.00 ± 0.00	1000.00 ± 0.00	500.00 ± 0.00	1000.00 ± 0.00	500.00 ± 0.00	666.67 ± 288.68	1000.00 ± 0.00	833.33 ± 288.68	1000.00 ± 0.00
**Resveratrol**	>1000.00	>1000.00	>1000.00	>1000.00	>1000.00	>1000.00	1000.00 ± 0.00	>1000.00	>1000.00	>1000.00
***Trans*-** ** cinnamaldehyde **	500.00 ± 0.00	500.00 ± 0.00	500.00 ± 0.00	500.00 ± 0.00	500.00 ± 0.00	500.00 ± 0.00	500.00 ± 0.00	833.33 ± 288.68	500.00 ± 0.00	500.00 ± 0.00

*Salmonella* strains were isolated from poultry.

## Data Availability

The original contributions presented in this study are included in this article; further inquiries can be directed to the corresponding author.

## Supplementary Materials

The following supporting information can be downloaded at: https://www.mdpi.com/article/10.3390/biology15141141/s1, Table S1: Individual data obtained for MIC and MBC values obtained for all compounds and strains tested; Table S2: Antibiogramas cepas LIST y SALM (10+10).

## Abbreviations

The following abbreviations are used in this manuscript:

AMC	Amoxicillin-clavulanic acid
AMP	Ampicillin
ATCC	American Type Culture Collection
ATP	Adenosine triphosphate
AZM	Azithromycin
C	Chloramphenicol
CAPE	Caffeic acid-phenethyl ester
CFU	Colony forming unit
CIP	Ciprofloxacin
CLSI	Clinical and Laboratory Standards Institute
CN	Gentamicin
CRO	Ceftriaxone
E	Erythromycin
EEP	Ethanolic extracts of propolis
EFSA	European Food Safety Authority
EU	European Union
EUCAST	European Committee on Antimicrobial Susceptibility Testing
FDA	United States Food and Drug Administration
GRAS	Generally Recognized as Safe
IMP	Imipenem
IPC	Isoprenyl caffeate
MBC	Minimum bactericidal concentration
MEM	Meropenem
MIC	Minimum inhibitory concentration
MRL	Maximum residue level
OCLA	Oxoid Chromogenic *Listeria* Agar
P	Penicillin
RASFF	Rapid Alert System for Food and Feed
RD	Rifampicin
STCC	Spanish Type Culture Collection
SXT	Trimethoprim/sulfamethoxazole
TE	Tetracycline
TSA	Tryptone soy agar
TSB	Tryptone soy broth
USA	United States of America
VA	Vancomycin
YOPI	The young, the old, the pregnant, and the immunocompromised
